# UHPLC-HRMS-based metabolomic and lipidomic characterization of glioma cells in response to anlotinib

**DOI:** 10.1038/s41598-023-34902-5

**Published:** 2023-05-17

**Authors:** Yingying Shi, Zhuolun Li, Qiuzheng Du, Wenxi Li, Jiyun Liu, Qingquan Jia, Lianping Xue, Xiaojian Zhang, Zhi Sun

**Affiliations:** 1 Department of Pharmacy, the First Affiliated Hospital of Zhengzhou University, Zhengzhou, 450052 Henan Province People’s Republic of China; 2 Henan Engineering Research Center of Clinical Mass Spectrometry for Precision Medicine, Zhengzhou, 450052 Henan Province People’s Republic of China; 3 Department of Pharmacy, Zhengzhou Traditional Chinese Hospital of Orthopaedics, Zhengzhou, 450052 Henan Province People’s Republic of China; 4 School of Pharmaceutical Sciences, Xiamen University, Xiamen, 361102 Fujian Province People’s Republic of China

**Keywords:** Lipidomics, Metabolomics

## Abstract

Anlotinib, as a promising oral small-molecule antitumor drug, its role in glioma has been only reported in a small number of case reports. Therefore, anlotinib has been considered as a promising candidate in glioma. The aim of this study was to investigate the metabolic network of C6 cells after exposure to anlotinib and to identify anti-glioma mechanism from the perspective of metabolic reprogramming. Firstly, CCK8 method was used to evaluate the effects of anlotinib on cell proliferation and apoptosis. Secondly, ultra-high performance liquid chromatography-high resolution mass spectrometry (UHPLC-HRMS)-based metabolomic and lipidomic were developed to characterize the metabolite and lipid changes in cell and cell culture medium (CCM) caused by anlotinib in the treatment of glioma. As a result, anlotinib had concentration-dependent inhibitory effect with the concentration range. In total, twenty-four and twenty-three disturbed metabolites in cell and CCM responsible for the intervention effect of anlotinib were screened and annotated using UHPLC-HRMS. Altogether, seventeen differential lipids in cell were identified between anlotinib exposure and untreated groups. Metabolic pathways, including amino acid metabolism, energy metabolism, ceramide metabolism, and glycerophospholipid metabolism, were modulated by anlotinib in glioma cell. Overall, anlotinib has an effective treatment against the development and progression of glioma, and these remarkable pathways can generate the key molecular events in cells treated with anlotinib. Future research into the mechanisms underlying the metabolic changes is expected to provide new strategies for treating glioma.

## Introduction

Glioma is the most common and lethal primary brain tumor, accounting for 81% of central nervous system malignancies^1^. Higher grade glioma is highly invasive and recurrent, with a median survival of 15 months^2^. The high incidence of the glioma occurs in young and middle-aged adults, bringing heavy economic burden to society and families. Currently, radio-chemotherapy followed by surgical resection is the standard treatment strategy in glioma patients^3^. However, due to the biological characteristics of unclear boundary between glioma and normal brain tissue, surgical treatment is difficult to complete resection. The postoperative chemotherapy plays an important role in further killing the residual glioma cells. Nevertheless, it is serious that the alkylating agent temozolomide is the preferred chemotherapy drug recommended for the treatment of malignant glioma, but the increasing incidence of drug resistance and long-term complications caused by drugs cannot be ignored^4^. Therefore, it is urgent to develop new therapeutic drugs and study multiple combined therapy.

Anlotinib is a novel multi-target tyrosine kinase inhibitor, which can effectively inhibit VEGFR, PDGFR, c-kit and other kinases^5^. It has shown good safety and efficacy in a variety of solid tumors such as non-small cell lung cancer (NSCLC), soft tissue sarcoma, and renal cell carcinoma^6^. As a promising oral small-molecule antitumor drug, its role in glioma has been reported in a small number of case reports. Recently, a new study found that anlotinib inhibited proliferation, invasion and migration of glioblastoma cells^7^. Interestingly, it also found that tumor supernatant from glioblastoma cells treated with anlotinib inhibited angiogenesis in human umbilical vein endothelial cells. Therefore, we particularly focused on the roles of anlotinib-mediated metabolic reprogramming in glioma cell.

Since the discovery of isocitrate dehydrogenase 1 (IDH1) mutation in glioma^8^, metabolic abnormalities have attracted more and more attention in tumor. Metabolic reprogramming in tumor microenvironment is an emerging hallmark of solid cancers. Studies have shown that metabolic reprogramming of glioma cells can lead to a large number of abnormal metabolites^9,10^. These metabolites not only play an important role in the proliferation and other biological behaviors of glioma cells, but also reshape their tumor microenvironment. With the in-depth study of metabolic reprogramming of glioma, it is noted that the enhancement of fatty acid oxidation can improve the metabolic plasticity of glioma cells, so as to make glioma cells adapt to the dynamic changes of metabolic reprogramming^11^. Therefore, this study will deeply understand the regulatory effect of anlotinib on the metabolic abnormalities of glioma cells from the perspective of metabolic reprogramming.

In this study, CCK8 method was used to explore the effect of anlotinib on cell viability and its cytotoxicity. Secondly, untargeted metabolomic was used to evaluate the changes of cell and cell culture medium (CCM) metabolism caused by anlotinib in the treatment of glioma. Untargeted metabolomic is a method to study the overall changes of small molecule metabolites, which reflects the physiological activities in organisms^12^. This technology has been applied to study the toxic mechanism of external stimuli by measuring the metabolic changes in organisms. Then, the effect of anlotinib on lipid metabolism of glioma cells and CCM was further studied by lipidomic. Lipidomic is a branch of metabolomics and a new method to provide a complete interpretation of lipid changes^13^. This study is the first in vitro experiment to preliminarily study the inhibitory effect of anlotinib on glioma cells from the aspect of metabolic reprogramming, suggesting that the anlotinib may become a new choice for glioma. Understanding the metabolic reprogramming of cancer and studying the mechanism of metabolic changes in cancer is expected to provide a new strategy for the treatment of glioma.

## Materials and methods

### Reagents and drugs

F12k culture medium, phosphate buffer saline (PBS), Penicillin–streptomycin double antibody and normal saline were procured from Servicebio (Wuhan, China). Fetal bovine serum and 0.25% trypsin–EDTA were purchased from Gibico (Waltham, MA, USA). Dimethyl sulfoxide was obtained from Macklin (Shanghai, China). HPLC-grade methanol, acetonitrile, formic acid and MS-grade ammonium acetate were procured from Fisher Scientific. Dichloromethane was obtained from Merck (Darmstadt, Germany). Standards of analytes and internal standards were derived from Sigma Aldrich (St. Louis, MO, USA). Anlotinib was obtained from Chia Tai Tianqing Pharmaceutical Group Co.,Ltd. The drug was dissolved in dimethyl sulfoxide (DMSO) for preparation of stock solution, where the final concentration of DMSO in the drug working solution in the cells was < 0.01%. DMSO of 0.01% was used as a vehicle control in all cell culture assays.

### Cell culture and viability assay

C6 cell line was procured from the American Type Culture Collection (ATCC; Manassas, VA, USA) and routinely cultured in F12K medium containing 10% fetal bovine serum and 1% penicillin/streptomycin at 37℃ in a 5% CO_2_ . The medium was replaced every 2 days until confluency. Cell inhibition was evaluated using Cell Counting Kit-8 assay (CCK8, Dojindo, Japan) according to the manufacturer’s instructions. Cells were seeded in 96-well plates at a density of 1 × 10^4^ cells/well and incubated for 24 h. Then C6 cells were treated with different concentration of anlotinib (0, 1, 5, 10, 25, 50 μM) for 24, 48, 72 h, respectively. Afterwards, 20 μL CCK8 reagents was added to each well and incubated for 1 h. The absorbance value was measured at a wavelength of 450 nm. All experiments were repeated for three times. A representation of cellular activity was as follows: Cell Viability (%) = (OD _drug_ − OD _blank_)/(OD _control_ − OD _blank_) × 100.

### Sample preparation

Cells were seeded on 6-well plates at a density of 1 × 10^7^ cells/well. At 80% of confluency, C6 cells were treated with anlotinib based on cell viability results for 24 h. Cells treated with PBS served as controls. Six duplicates were prepared for each group. Metabolite and lipid extraction was performed using a modified Bligh-Dyer extraction method^14^.

#### CCM metabolite and lipid extraction

The culture medium was aspirated, and then the methanol/dichloromethane/water (3:3:2, v/v/v) was added to the sample at a volume of (1:4, v/v) and vortexed for 5 min. The resulting two-phase solution was equilibrated for 5 min and then centrifuged at 13,000 rpm for 10 min. The upper and lower layers were separated into new tubes and dried under N_2_. After that, the upper layer was redissolved in 80% methanol for metabolomic analysis and the lower was redissolved in isopropanol for lipidomic analysis.

#### Cell metabolite and lipid extraction

Cells were washed twice with pre-chilled normal saline, trypsinized, and washed again with PBS. Count about 1 × 10^7^ cells as a sample and centrifuged at 1500 rpm for 5 min. After removing the supernatant, five sample volumes of the methanol/dichloromethane /water (3:3:2, v/v/v) previously stored at -40℃ were added to the cell. Afterwards, the samples were subjected to three freeze–thaw (-80 ℃/room temperature) cycles and broken with an ultrasonic cell crusher under ice bath conditions. The samples were vortexed for 30 s and equilibrated for 10 min, and centrifuged at 13,000 rpm for 10 min. After centrifugation, the two-phase solutions were collected separately and dried under N_2_. Finally, the upper extracts were reconstituted in 80% methanol for metabolomic analysis and the lower was redissolved in isopropanol for lipidomic analysis.

### LC–MS for untargeted metabolomic analysis

Untargeted metabolomic was performed on ultra-high performance liquid chromatography- high resolution mass spectrometry (UHPLC-HRMS). Chromatographic separation was performed on an Dionex Ultimate 3000 UHPLC (Thermo Fisher Scientific, USA) system using an ACQUITY UPLC BEH C18 (1.7 um, 2.1 mm × 50 mm, Waters) column kept at 40 ℃. The mobile phase was composed of water with 0.1% formic acid (solvent A) and acetonitrile (solvent B). The elution gradient was as follows: 0 ~ 2 min, 5%B; 2 ~ 15 min, 5% ~ 100% B; 15 ~ 18 min, 100%B. The flow rate was maintained at 0.3 mL/min. The sample injection volume was 5 μL.

Detection was carried out on a Q-Exactive Orbitrap mass spectrometer (Thermo Fisher Scientific, USA) with autosampler and online vacuum degasser. The mass spectra were acquired through Full MS/ddms^2^ scan patterns operated in positive and negative ion mode. For Full MS scan pattern: resolution, 70 000 FWHM (full width half maximum); automatic gain control (AGC) target, 3 × 10^6^ ions; maximum injection time: 100 ms. For ddms^2^ scan pattern: resolution: 17 500 FWHM; AGC target: 1 × 10^5^ ions; loop count: 5; maximum injection time: 50 ms; isolation window: 2.0 m/z. Other detail parameters: mass range, 80–1200 m/z; S-lens RF level, 50; sheath gas pressure, 40 arb; aux gas pressure, 10 arb; heater temperature, 300 °C; capillary temperature, 320 °C; spray voltage, + 3500 V and − 3200 V; collision energies, 20, 40 and 60 eV.

Quality control (QC) samples were prepared by pooling equal volumes (20 μL) of all cell samples and CCM samples, respectively. Three parallel pooled QC samples were continuously injected into the instrument prior to analysis, and the QC samples were inserted into every 5 tested samples during the acquisition so as to assess the stability of UHPLC -HRMS system.

### LC–MS for untargeted lipidomic analysis

Untargeted lipidomic was carried out on Thermo Scientific Dionex Ultimate 3000 UHPLC system coupled to hybrid quadrupole-Orbitrap high-resolution mass spectrometer equipped with a heated electrospray ionization probe (HESI). The samples were separated on an ACQUITY UPLC CSH C18 column (1.7um, 2.1 mm × 100 mm, Waters) at 40 °C with a constant flow rate of 0.30 mL/min. The sample injection volume was 5 μL. The mobile phase A was acetonitrile/water (6:4, v: v) and the mobile phase B was acetonitrile/ isopropanol (1:9, v: v), both containing 10 mM ammonium formate. The gradient was set as follow: 0–2 min, 30%B; 2–20 min, 30%–100% B; 25–30 min, 100% B.

After separation, high-resolution MS data were acquired on a Q-Exactive Orbitrap mass spectrometer (Thermo Fisher Scientific, USA) in positive and negative ion mode. The detail parameter settings were as follows: heater temperature, 300 °C; capillary temperature 350 °C, spray voltage + 3.5 kV for positive ion mode and − 3.2 kV for negative ion mode; sheath gas flow rate, 40 arb; aux gas flow rate, 10 arb; sweep gas flow rate, 0 arb; loop count, 5; isolation window, 2.0 m/z; S-lens RF level, 50; collision gas and dissociation, nitrogen; collision energy, 20, 40, 60 eV. Lipidomic profiles were acquired in the m/z 80–1200 range with a resolution of 70,000 in full-scan pattern and 17,500 in ddms^2^ scan pattern.

QC samples were generated by pooling equal volumes of each samples tested. One QC sample was injected in every five samples tested to assess and correct for retention time shifts, relative standard deviations (RSD) of peak areas and mass accuracy.

### Data processing

The original MS data was acquired with XcaliburTM 3.0 software (Thermo Scientific, USA). Metabolomic and lipidomic data were preprocessed by Compound Discoverer 3.0 software and LipidSearch 4.2 software (Thermo Fisher Scientific, USA), respectively, for peak picking, peak alignment, baseline correction and peak area normalization. Correction for batch effects was carried out using a univariate approach^15^ namely quality control–based robust LOESS (locally estimated scatterplot smoothing) signal correction (QC-RLSC).

For the metabolomic analysis, the metabolic features identification must meet three strict criteria as previously described^16–18^: narrow window retention (0.2 min), accurate mass with variation less than 5 ppm and MS/MS spectra patterns. Firstly, the metabolites were identified by comparing the exact molecular mass, retention time (RT) and MS^2^ fragment of the peak with data from standard materials and in-house metabolite library. Second, further metabolites were identified by analyzing the mass spectrometry cleavage pattern, mass error and further confirming the chemical structure by comparison with public mass spectrometry databases including mzCloud database (https://www.mzcloud.org/), Human Metabolome Database (HMDB, https://hmdb.ca/), ChemSpider database (http://www.chemspider.com/) and Masslist (Thermo Compound Discoverer internal database for endogenous metabolites).

For the lipidomic analysis, the lipid features identification was based on the accurate mass of precursor ions and the MS^2^ spectral pattern. The detailed parameter settings were as follows: precursor tolerance, 5 ppm; product tolerance, 5 ppm; product ion intensity threshold, 5%; m-Score threshold, 2; Quan m/z tolerance, ± 5 ppm; Quan retention time range, ± 1.0 min; use of all isomer peaks filter and ID quality filters A, B and C; Adduct ions: + H and + NH4 for positive ion mode, and − H and + HCOO for negative ion mode; alignment method, mean.

A data matrix containing the molecular weight, retention time and peak area of the metabolites and lipids were obtained, respectively. Subsequently, the data matrix was imported into SMICA 14.1 software for principal component analysis (PCA), which was used to indicate the degree of aggregation and dispersion between groups. Furthermore, heatmaps of differential metabolites were drawn using MeV software, pathway enrichment analysis was performed using MetaboAnalyst 3.0 online software, and histogram was conducted using GraphPad Prism 8.0.1.

### Statistical analysis

Statistical difference among groups was analyzed using SPSS 24.0 software (IBM, USA) and GraphPad Prism 8.0.1 software (GraphPad Software Inc, San Diego, United States). Differential analysis was performed using a student t test. *P* values were adjusted for multiple hypothesis using the Benjamini–Hochberg method and analytes with false discovery rate (FDR) below 0.05 were considered significant.

## Results

### The effect of anlotinib on cell viability

**Figure 1:**
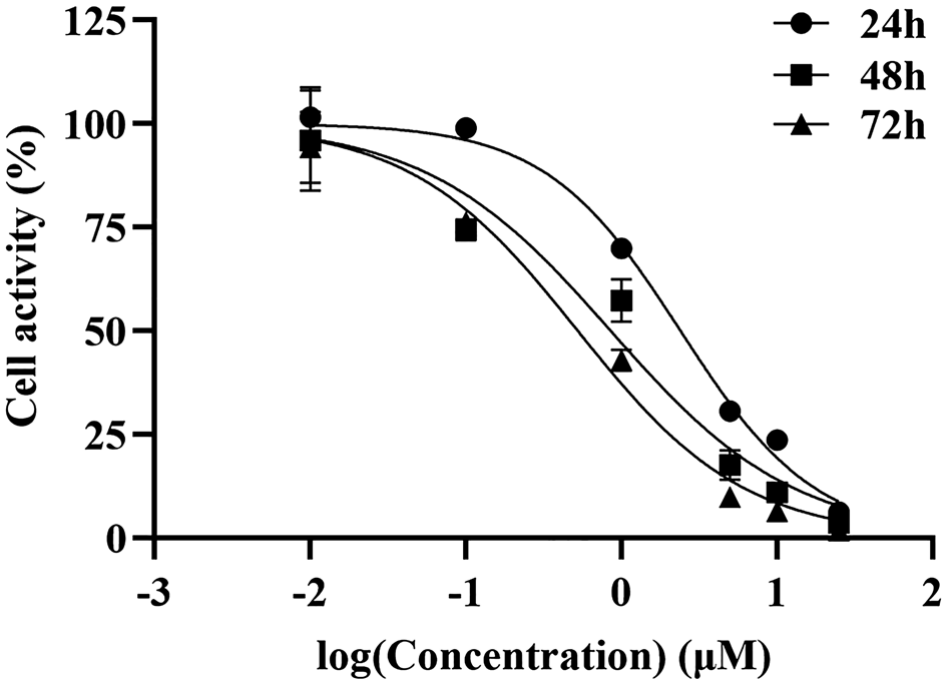
Effects of anlotinib on C6 proliferation under different concentrations.

To assess the impact of anlotinib on cell viability, C6 cells were exposed to different dose of anlotinib. As shown in Fig. 1, the cell viability of C6 decreased with the increase of anlotinib concentration, indicating that anlotinib had concentration-dependent inhibitory effect with the set concentration range. The IC50 value of anlotinib for 24 h was 2.396 μM. According to previously published work^19^, the concentration equivalent to 80% survival rate (1 μmoL/L) was selected for further experiments.

### LC–MS Profiling and Methodological evaluation results

**Figure 2:**
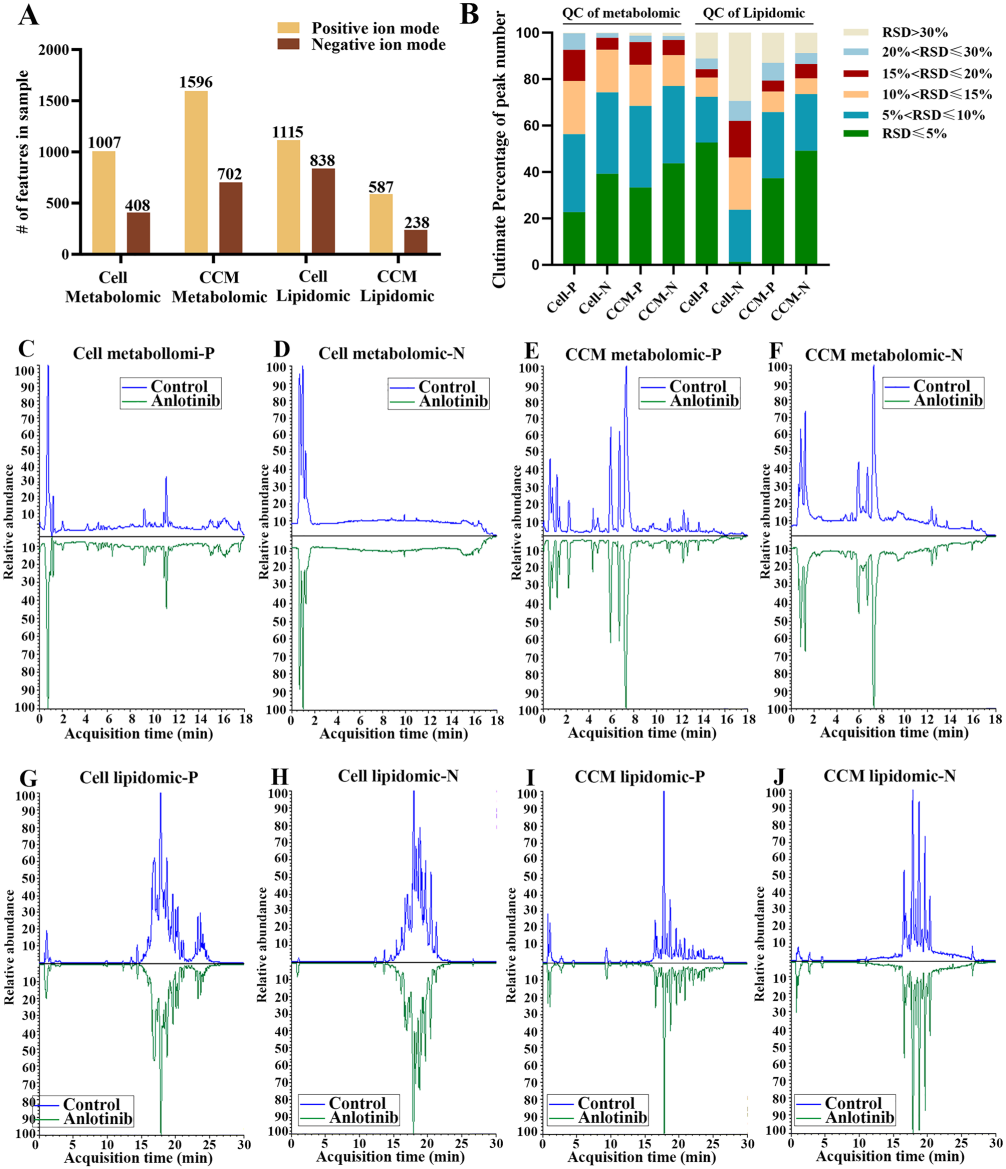
Methdological evaluation results and representative typical total ion chromatograms of sample in each group. (**A**) The cumulative number of detected metabolic and lipid features in each group. (**B**) RSD distribution of peak ion features in QC samples. RSD, relative standard deviation. Representative TICs of cell (**C**, **D**) and CCM (**E**, **F**) sample based on metabolomic analysis in positive and negative ion mode, respectively. Representative TICs of cell (**G**, **H**) and CCM (**I**, **J**) sample based on lipidomic analysis in positive and negative ion mode, respectively. TICs, typical total ion chromatograms; CCM, cell culture medium.

The metabolites and lipids were analyzed by UHPLC-HRMS in both positive and negative ion modes. Global metabolomics analysis on control and anlotinib samples generated 1007 (+) and 408 (−) features in cell, and 1596 (+) and 702 (−) features in CCM. For lipidomic analysis, a total of 1115 (+) and 838 (−) features were detected in cell, and 587 (+) and 238 (−) features were covered in CCM, respectively (Fig. 2A). Their representative typical total ion chromatograms (TICs) were presented in Fig. 2C-2J. After repeated detecting of QC samples, relative standard deviation (RSD) value of each feature was calculated according to the peak area in QC samples (Fig. 2B). In positive and negative ion mode, the peak accumulative proportion of metabolites with RSD less than 30% was 99.70 and 99.75 in cell metabolomic analysis, 98.75 and 98.58 in the CCM metabolomic analysis, 88.88 and 70.64 in the cell lipidomic analysis, 87.05 and 91.18 in the CCM lipidomic analysis. These results showed that peak features with RSD less than 30% accounted for more than 70%, indicating that the metabolomic and lipidomic method have good reliability and high quality of data.

### Metabolic changes induced by anlotinib in C6 Cells

**Figure 3:**
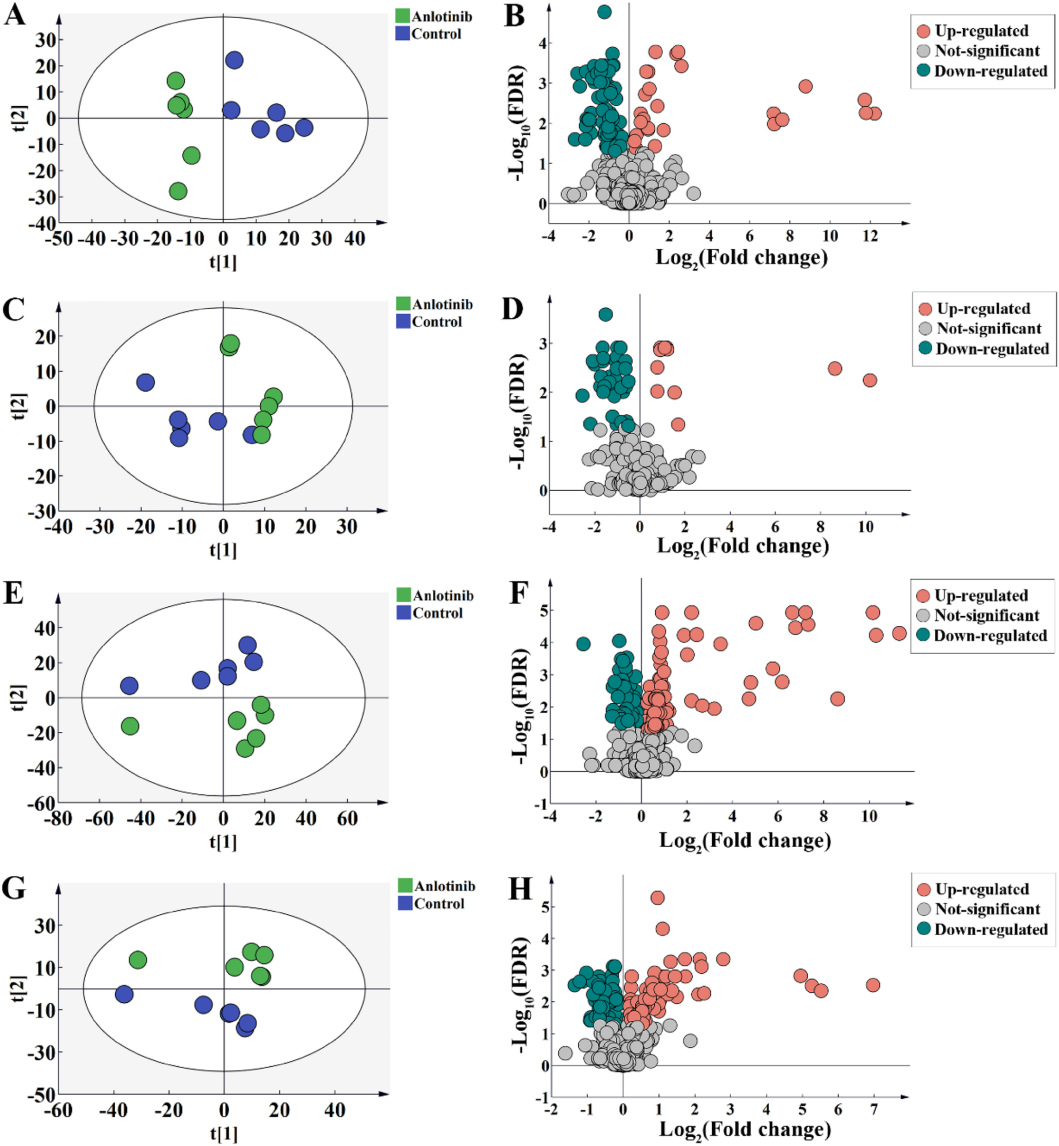
Metabolomic analyses reveal metabolic responses to anlotinib. PCA score plot and volcano plots for cell metabolites detected in positive (**A**, **B**) and negative (**C**, **D**) ion mode, respectively. PCA score plot and volcano plots for CCM metabolites detected in positive (**E**, **F**) and negative (**G**, **H**) ion mode, respectively. Volcano plot showing metabolites from anlotinib treatment groups vs control groups with various fold changes and *P*-values. Metabolites that pass the cutoff *P* values corrected for false discovery rate < 0.05 are depicted in green or red, respectively. PCA, principal component analysis; CCM, cell culture medium.

**Figure 4:**
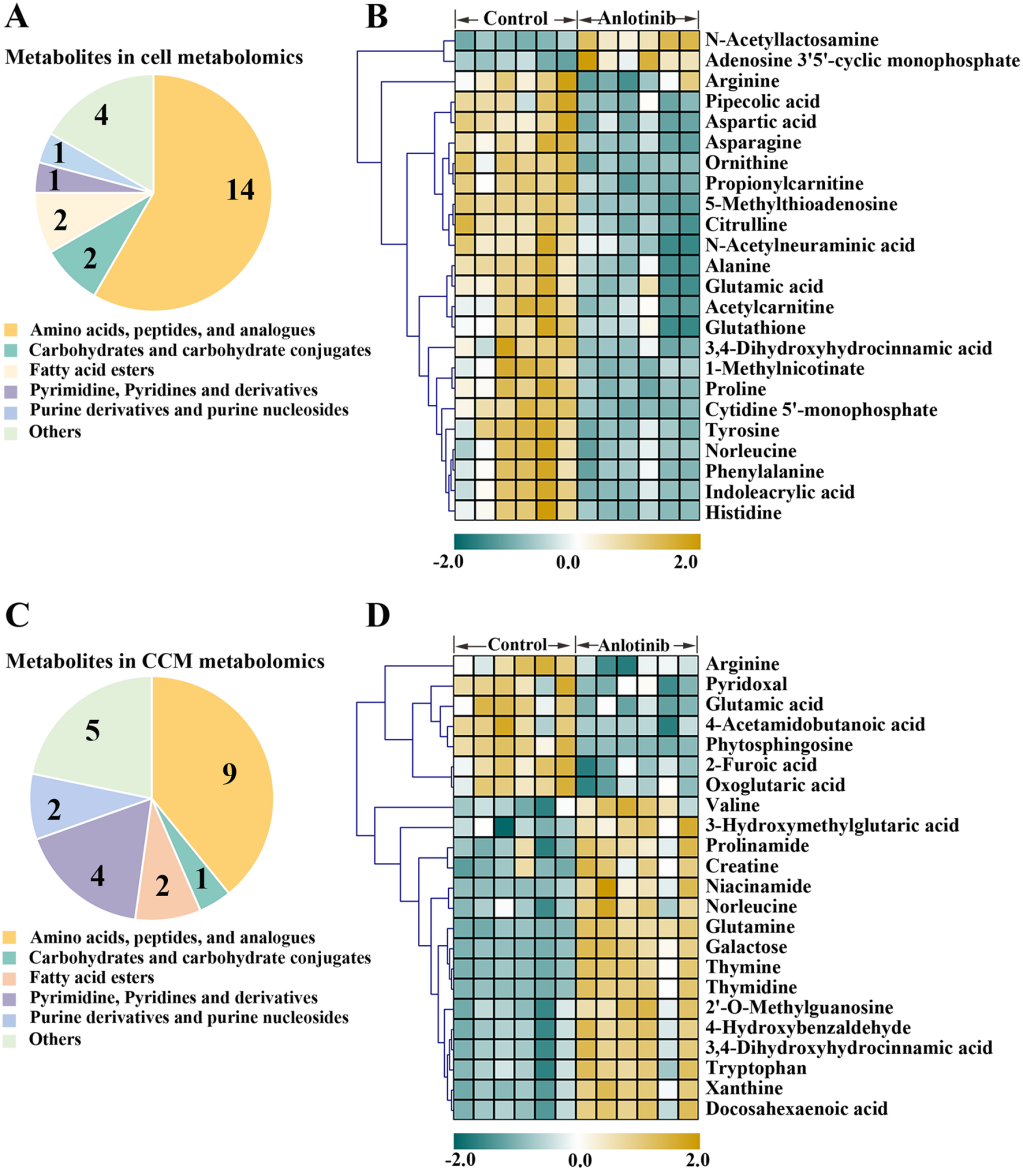
The categories and changes of differential metabolites. The distribution of annotated metabolites in cell (**A**) and CCM (**C**) metabolomic analysis, respectively. Heatmap analysis of the perturbed metabolites in cell (**B**) and CCM (**D**) metabolomic analysis, respectively. Significant up-and down-regulated metabolites were shown in yellow and green color, respectively. CCM, cell culture medium.

**Figure 5:**
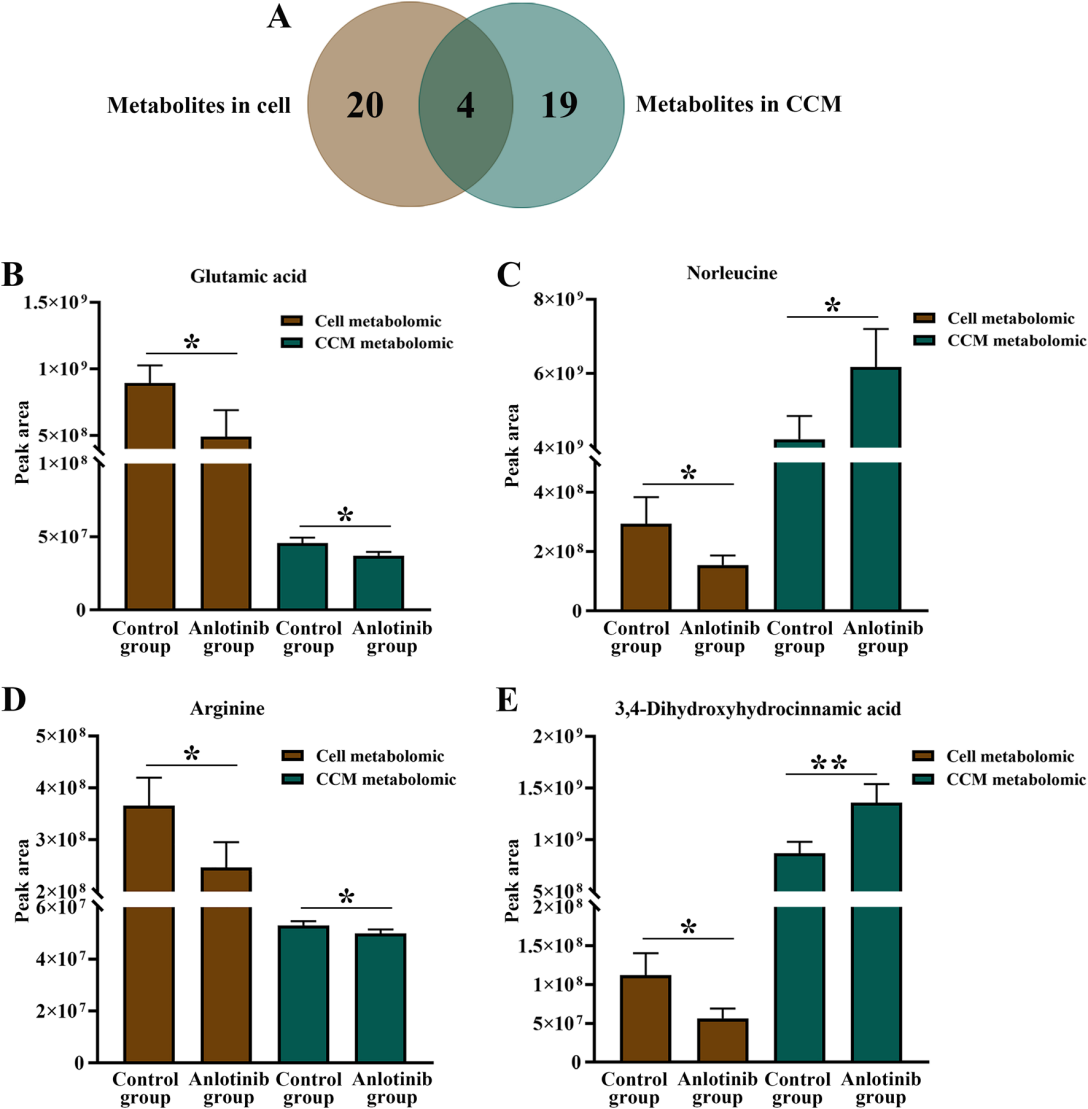
Four common differential metabolites screened in cell and cell culture medium metabolomics. *P < 0.05; **P < 0.01 versus control group.

**Figure 6:**
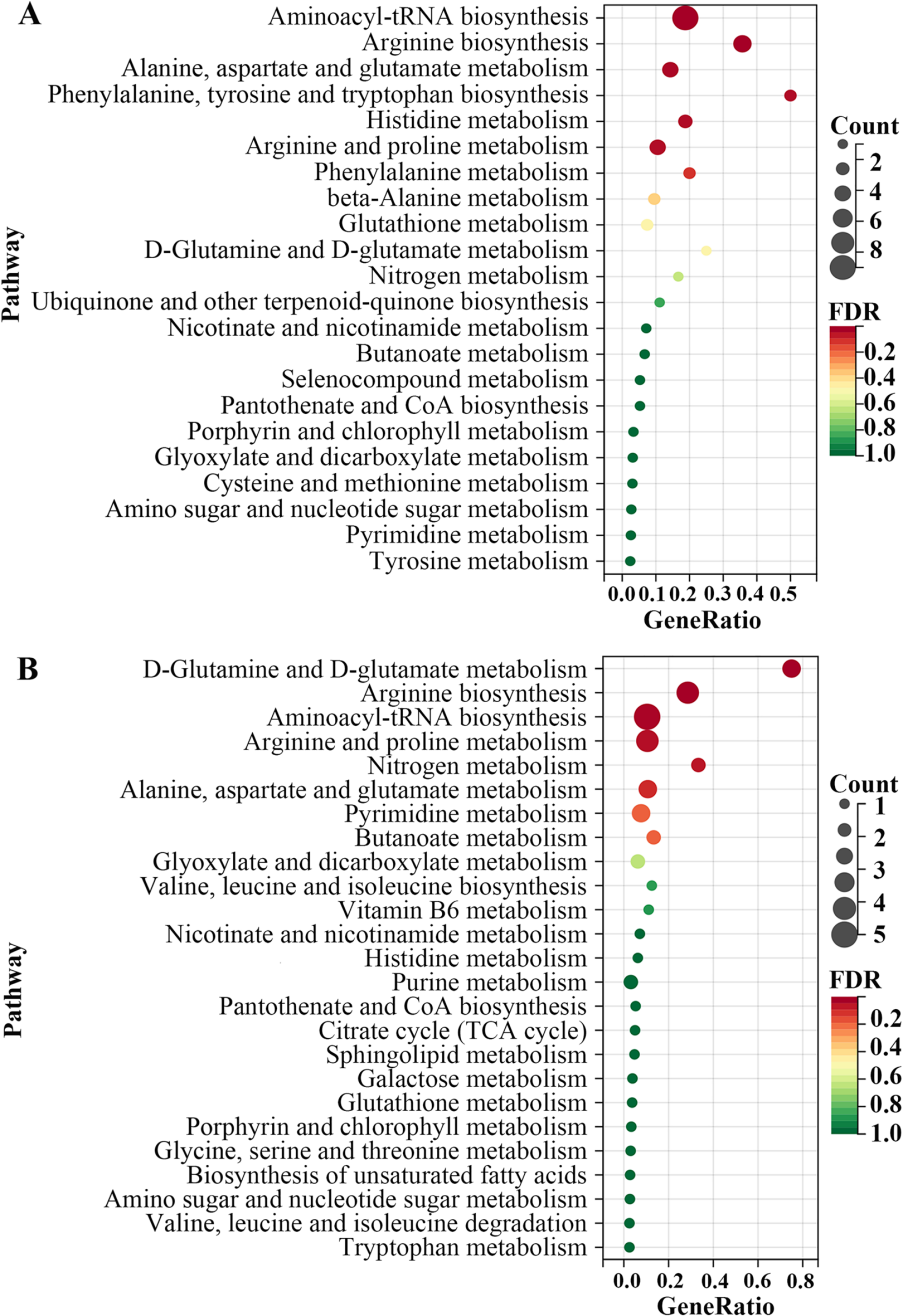
Pathway enrichment analysis on the basis of the altered cell (**A**) and cell culture medium (**B**) metabolites.

PCA score showed that there was significant separation between the anlotinib group and the control group, indicating that the intervention of anlotinib led to alterations in cell and CCM metabolites (Fig. 3). According to the criteria (*FDR* < *0.05*), a total of 24 and 23 disturbed metabolites were identified in cell and CCM respectively (Fig. 3 and Supplementary Table S1). In particular, anlotinib treatment resulted in significant perturbation of a large number of metabolites related to amino acid metabolism (Fig. 4A and C). As observed in the heatmap (Fig. 4B and D), the levels of the most of cell differential metabolites in the anlotinib group decreased, while the levels of most of the differential metabolites in the CCM treatment group increased. Differential metabolites were enriched in various biological pathways and the results showed that they mainly affected the metabolic pathways of arginine biosynthesis, arginine and proline metabolism, alanine, aspartate and glutamate metabolism (Fig. 5). Integrated analysis of cell and CCM differential metabolites revealed that there were four common metabolites, namely arginine, glutamic acid norleucine and 3,4-dihydroxyhydrocinnamic acid (Fig. 6).

### Lipidomic changes induced by anlotinib in C6 Cells

**Figure 7:**
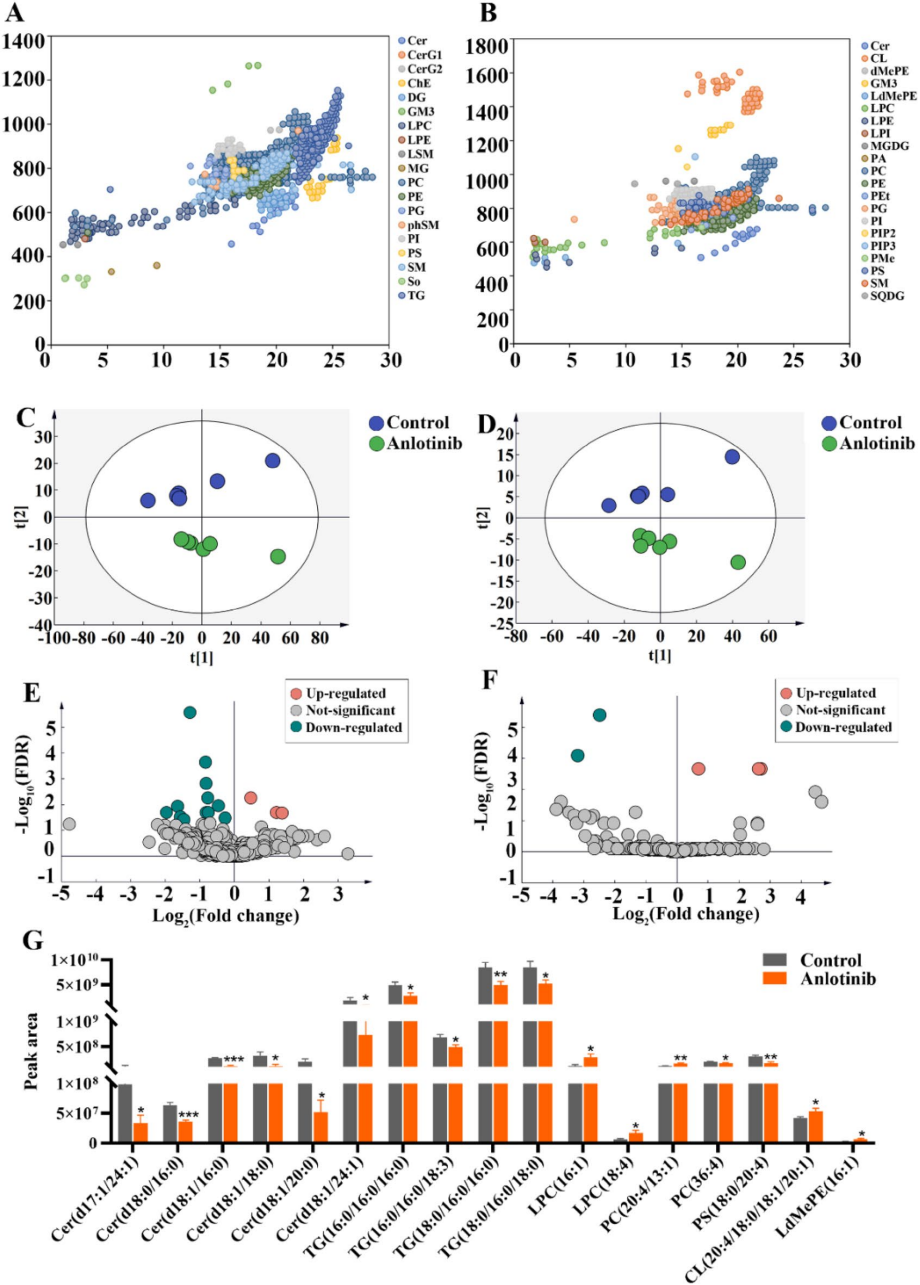
Expression of lipids involved in lipidomic analyses. Overview of detected lipids in positive (**A**) and negative ion mode (**B**). PCA score plots and volcano plots of lipidomic profiles with anlotinib treatments in positive (**C**, **E**) and negative (**D**, **F**) ion mode, respectively. Volcano plot showing lipids from anlotinib treatments groups vs control groups with various fold changes and *P*-values. Lipids that pass the cutoff *P* values corrected for false discovery rate < 0.05 are depicted in green or red, respectively. The histogram of differential lipids identified in lipidomic analysis (**G**). PCA, principal component analysis; Cer, ceramide; LPC, lysophosphatidylcholine; PC, phosphatidylcholine; PS, phosphatidylserine; TG, triglyceride; CL, cardiolipin; LdMePE, lysodimethylphosphatidylethanolamine.

PCA analysis was performed to obtain a comprehensive view of the clustering of each sample (Fig. 7A and B). It was found that the data of the treatment group and the control group had a significant trend of separation in cell, suggesting a disturbance in the lipid profiles after anlotinib intervention. However, there was no trend of separation in the lipidomic changes of CCM across the two groups. The cell lipidomic results showed that a total of 19 and 21 lipid subclasses were identified in positive and negative ion mode, respectively (Fig. 7C and D). According to the screening results (Fig. 7E and F), a total of 17 differential lipids in cell were identified, covering 7 lipid classes, namely lysophosphatidylcholine (LPC), phosphatidylcholine (PC), phosphatidylserin (PS), triglyceride (TG), cardiolipin (CL), ceramide (Cer), and lysodimethylphosphatidylethanolamine (LdMePE). The changes and specific information of various lipids were shown in Table S3. The predominant lipid class was Cer and TG. The histogram was conducted to visualize metabolic disparities for the 17 lipids with lowest *P*-values after FDR correction (Fig. 7G). The levels of Cer and TG in C6 cells were significantly downregulated by anlotinib.

### The function mechanism analysis of anlotinib on C6 cells

**Figure 8:**
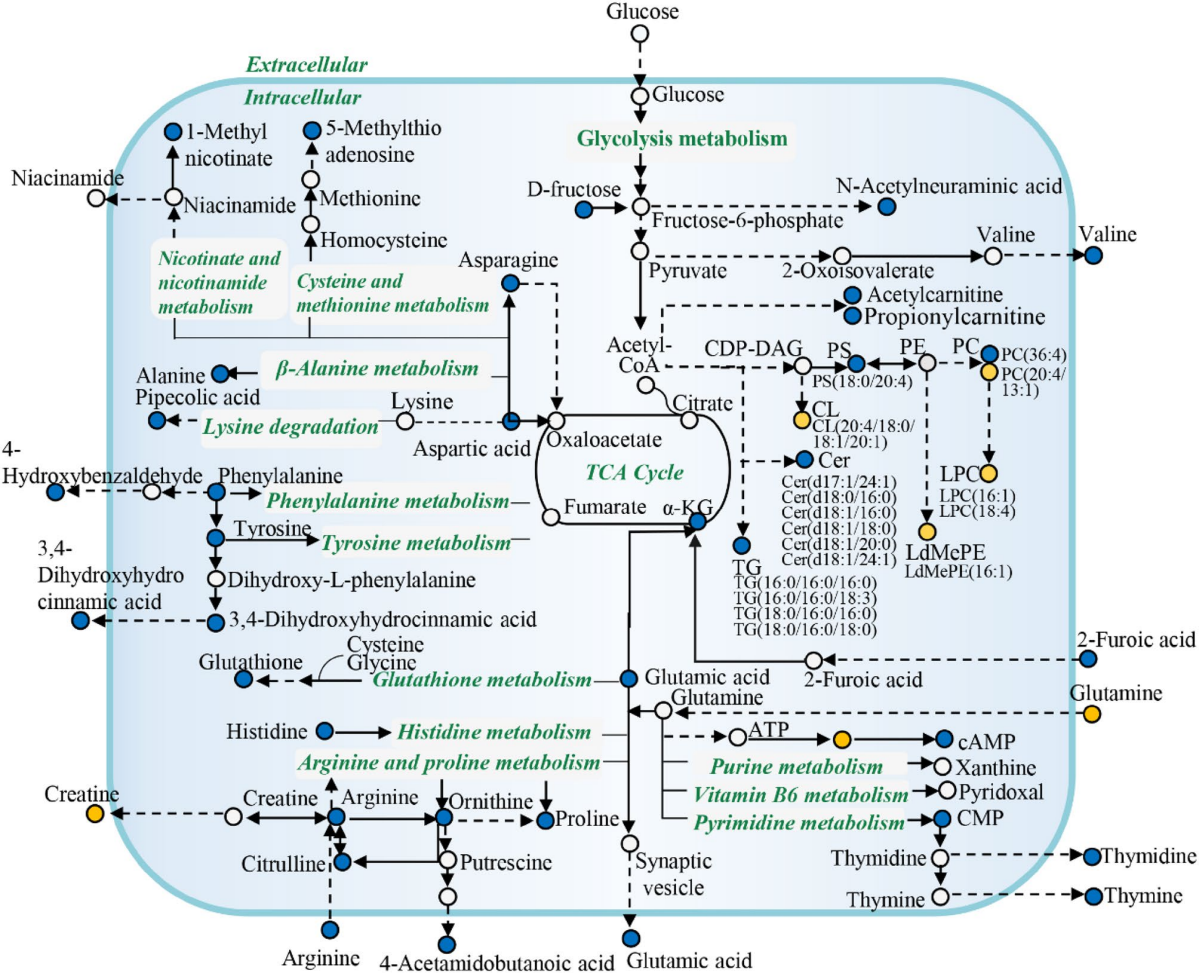
Metabolic network of glioma C6 cells after exposure to anlotinib. Molecules marked in orange and blue dots represented the up- and down-regulation, respectively.

To better understand the therapeutic mechanism of anlotinib against glioma, differential metabolites and lipids were annotated using the Kyoto Encyclopedia of Genes and Genomes (KEGG) database and then mapped to the KEGG pathway. The interactions of different metabolites and lipids in organisms form different metabolic network pathways. Following the annotation results, a metabolic pathway diagram was constructed to reflect the effect of anlotinib on the overall metabolic network (Fig. 8). The results provided a global profile of metabolic changes in response to the anlotinib treatment. Anlotinib mainly affected the amino acid metabolism, energy metabolism and lipid metabolism of glioma, which may be valuable for glioma treatment.

## Discussion

Metabolic adaptability is fundamental to cancer cell survival. On the one hand, the metabolic reprogramming of tumor cells is to meet the needs of rapid proliferation, and on the other hand, it is to compensate for the redox stress caused by the increased energy demand of tumor cells. Cell metabolomics focusing on specific cell types can provide a more stable background and reduce differences in metabolites caused by other factors, thus making subtle metabolite changes more apparent. By combining intracellular and extracellular metabolite profiling, a more complete biological explanation of the metabolic regulation of anlotinib can be provided. Here, our findings suggested that changes in the metabolic pattern of glioma C6 cells after anlotinib intervention were mainly reflected in amino acid metabolism and energy metabolism.

In this study, glutamate and glutathione tended to decrease in C6 cells treated with anlotinib. Glutamic acid promotes a series of metabolic reactions, including the synthesis of glutathione, cAMP, CMP, thymidine and thymine, all of which are important regulators of cell growth and survival. Glutamate is the main synthetic raw material of glutathione (GSH). Biochemical studies showed that glioblastoma (GBM) tumor cells rely on high levels of glutathione to maintain redox homeostasis in response to increased levels of redox stress under hypoxic conditions^20^. Several studies have reported that the increased GSH content was closely related to the higher drug resistance in GBM cells^21,22^. Accumulating evidence have revealed that glutamate and glutathione levels in brain tissue of high-grade gliomas were higher than those of low-grade gliomas^23^. In agreement with our results, the lower levels of glutamate and glutathione in the anlotinib group may reflect the insufficient amino acids for synthesizing glutathione after drug intervention, which further induces apoptosis of cancer cells.

As an important source of energy, glutamine can be converted into glutamate through deamination reaction catalyzed by glutaminase after entering the mitochondria, and then converted into α-ketoglutarate, thereby promoting the tricarboxylic acid cycle^24^. Compared with normal cells, glutamine was metabolized at higher levels in glioma cells^25^. In this study, after the intervention of anlotinib, the level of glutamine in the culture medium of C6 cells was significantly higher than that of the control group, which may be due to the influence of cell membrane transporters after administration, so that extracellular glutamine cannot enter tumor cells. Many lines of evidence suggested that the glutamine transporter (ASCT2/SLC1A5) was upregulated in C6 glioma cells^26^. Increased glutamine transporter expression is responsible for glutamine addiction in glioma cells^27^, so inhibition of glutamine transport is also considered as a potential new strategy for glioma therapy.

Meanwhile, the differential metabolites related to arginine and proline metabolism pathway, including arginine, ornithine, citrulline, proline and creatine, were downregulated in C6 cells after anlotinib intervention, which seriously affected the energy supply in cancer cells. In normal cells, arginine is synthesized from citrulline in the urea cycle by arginine succinate synthase 1 (ASS1) and arginine succinate lyase (ASL), and hydrolyzed by intracellular arginase for urea and ornithine^28^. Glioma cells take up more arginine from the extracellular environment by upregulating the cationic amino acid transporter 1 (CAT1/SLC7A1) to meet their proliferation and metabolic needs^29^. Reprogramming of arginine metabolism in GBM involves upregulation of CAT1 for arginine intake, upregulation of arginase for arginine catabolis, and downregulation of key enzymes involved in the endogenous arginine synthesis pathway^30^. Ornithine is a precursor of polyamine and proline biosynthesis and is required for various cellular functions. An earlier study incubated C6 GBM cell lines with different concentrations of proline and found that a significant increase in ROS and nuclear factor kappa B (NF-κB) activity was associated with an increase in the amount of proline, suggesting that proline may have a role in signal transduction of cell proliferation^31^. Current literatures found that the concentration of proline in GBM was higher than in normal brain^32^. Similarly, IDH-mutant high-grade oligodendrogliomas was found to utilize the proline cycle as a redox shuttle to maintain redox balance^33^. Our findings support the hypothesis that regulating the disorder of arginine and proline metabolism pathway in cancer cells can affect the proliferation of tumor cells, which provides a new idea for the targeted therapy of glioma.

Another notable change was a significant decrease of phenylalanine and tyrosine levels in C6 cells after exposure to anlotinib. Phenylalanine is one of the essential amino acids that cannot be synthesized by itself, so the culture medium is the main source of phenylalanine in cells. Phenylalanine has the function of regulating oxygen free radical scavenging, which have found that elevated levels of phenylalanine were associated with gliomas^34^. Phenylalanine can be oxidized to tyrosine by the catalysis of phenylalanine hydroxylase, which in turn is metabolized to fumarate. Tyrosine is a precursor substance for the synthesis of many neurotransmitters and important compounds in tumor angiogenesis and immunity. Recent studies have found that tyrosine metabolism was abnormal in glioma patients, and the expression of key metabolites such as phenylalanine and tyrosine in the blood plasma of patients with glioblastoma was upregulated compared with the healthy controls^35^. In addition, it was recently reported that the accumulation of tryptophan was also observed in GBM^36^. Collectively, therapeutic targets centered on glioma metabolism deserve more attention and in-depth research in the future.

Besides, we noticed that aspartic acid and asparagine were downregulated due to the anlotinib treatment. Aspartic acid, and asparagine are non-essential amino acids that are partially dependent on in *vivo* biosynthesis. In tumor cells, aspartate and asparagine not only participate in tumor cell proliferation, but also regulate signal transduction in cancer cells. Asparagine synthase (ASNS) is the key enzyme in asparagine synthesis. A growing body of literature indicates that the high expression of ASNS is associated with the glioma cell invasion^37^. Glutamine is also the main amino donor for asparagine synthesis. In the case of insufficient glutamine in tumor cells, exogenous supplementation of asparagine can maintain protein translation and promote tumor cell growth^37^. The intrinsic relationship between amino acid depletion and anlotinib treatment needs further research to provide a new entry point for the treatment of glioma.

Glioma is also characterized by excessive synthesis and utilization of lipids. The detected differential lipid components were of various types and complex structures, including Cer, TG and phospholipid. In this study, it was found that the level of multiple Cer in glioma cells decreased significantly after anlotinib intervention. Cer with different chain lengths have opposite biological functions in cell proliferation and apoptosis. Previous research has shown there were decreased levels of C18-ceramide in glioma^38^. However, there were also studies confirming that exogenous ceramides do not cause synergistic effects with irradiation or alkylating chemotherapy in glioma cells^39^. This suggested that regulation of intrinsic ceramide levels in glioma cells was insufficient to overcome resistance to standard glioma treatments. Cer plays a key central role in cell sphingolipid metabolism. Earlier studies have found a unique metabolic vulnerability in the sphingolipid pathway of gliomas with IDH1 mutations^40^. Therefore, the effect of anlotinib on glioma in regulating ceramide levels needs to be further explored. Another main chemical class with strong correlations with the glioma were TG. Previously, highly expression of TG and diacylglycerol-acyltransferase (DGAT1) have been observed in tissue samples from glioblastoma patients, revealing that DGAT1 protects glioblastoma cells from toxic damage caused by lipid metabolism imbalance by converting excess fatty acids into TG and storing them in lipid droplets^41^. In the lipid profiling analysis, we identified the content of TGs in glioma C6 cells were reduced after the intervention of anlotinib. Meanwhile, anlotinib affects glioma phospholipid metabolism, including PC, PS, LPC, CL and LdMePE. Abnormal expressed lipids can regulate a range of functions by participating in the formation and signal transduction process of tumor cells. Hence, focus on the study of lipid regulation mechanisms in order to find new cancer therapeutic targets and strategies.

Notably, our study has some challenges and limitations. Firstly, although we captured that C6 glioma cells exhibited significant metabolic effects after anlotinib intervention, we cannot rule out that there may be differences in cell-to-cell responses. Secondly, this study is exploratory and preliminary, the specific mechanism of how these metabolites and lipids are affected by anlotinib remains unclear, and further functional studies are needed to clarify. Nevertheless, we have taken an important first step in investigating metabolites associated with the efficacy of anlotinib in glioma treatment. Thirdly, the various AATs are both independent and interact with each other, and targeting one AAT may lead to inhibition or overexpression of the others. Therefore, many factors should be considered comprehensively in anti-tumor research. Finally, direct extrapolation of results from cell culture research to clinical practice may contain potential biases, but is still instructive.

## Conclusions

In summary, UHPLC-HRMS-based metabolomic and lipidomic method was first employed to reveal the potential metabolic regulatory mechanism of anlotinib on glioma C6 cells. Metabolomics analysis demonstrated that anlotinib induced alterations of 24 and 23 differential metabolites in cell and CCM respectively, which were closely related to arginine and proline metabolism, alanine, aspartate and glutamate metabolism, and amino acid metabolism. Further lipidomic profiling showed that anlotinib exposure caused significant changes in 17 lipids including glycerides, glycerophospholipids and sphingolipids. Collectively, the results provided a global profile of metabolic changes in response to the anlotinib treatment, and these remarkable pathways can produce the key molecular events in anlotinib-treated cells. Future research into the mechanisms underlying the metabolic changes is expected to provide new strategies for treating glioma.

## Supplementary Information

Supplementary Information.

## Data Availability

The datasets generated during and/or analysed during the current study are available from the corresponding author on reasonable request.
