# Supplementary material for: UHPLC-HRMS-based metabolomic and lipidomic characterization of glioma cells in response to anlotinib
Source: Sci Rep. 2023 May 17;13:8044. doi: 10.1038/s41598-023-34902-5 (PMC10192199; doi:10.1038/s41598-023-34902-5)
Supplement: Supplementary file 1 — Supplementary Information. [file 41598_2023_34902_MOESM1_ESM.docx]

Table S1 Differential metabolites identified in cell metabolomic analysis

| No. | Metabolite | Ion mode | | | Formula | Molecular Weight | | RT (min) | Fold change | | *P* value | | FDR | Class |
| --- | --- | --- | --- | --- | --- | --- | --- | --- | --- | --- | --- | --- | --- | --- |
| 1 | 5-Methylthioadenosine* | | P | C_11_H_15_N_5_O_3_S | | | 297.08957 | 0.66 | 0.38 | 1.69E-08 | | 1.70E-05 | | Others |
| 2 | Ornithine* | | P | C_5_H_12_N_2_O_2_ | | | 132.09001 | 0.67 | 0.17 | 1.16E-05 | | 5.82E-04 | | Amino acids, peptides, and analogues |
| 3 | Pipecolic acid | | P | C_6_H_11_NO_2_ | | | 129.07912 | 0.67 | 0.56 | 8.44E-04 | | 1.18E-02 | | Amino acids, peptides, and analogues |
| 4 | Arginine* | | P | C_6_H_14_N_4_O_2_ | | | 174.11184 | 0.71 | 0.68 | 2.41E-03 | | 2.50E-02 | | Amino acids, peptides, and analogues |
| 5 | Citrulline | | P | C_6_H_13_N_3_O_3_ | | | 175.09564 | 0.81 | 0.33 | 9.78E-06 | | 5.29E-04 | | Amino acids, peptides, and analogues |
| 6 | Alanine | | P | C_3_H_7_NO_2_ | | | 89.04772 | 0.81 | 0.45 | 5.29E-05 | | 1.55E-03 | | Amino acids, peptides, and analogues |
| 7 | Asparagine | | P | C_4_H_8_N_2_O_3_ | | | 132.05342 | 0.81 | 0.43 | 5.53E-05 | | 1.55E-03 | | Amino acids, peptides, and analogues |
| 8 | 1-Methylnicotinate | | P | C_7_H_7_NO_2_ | | | 137.04535 | 0.83 | 0.26 | 8.16E-04 | | 1.16E-02 | | Others |
| 9 | Glutamic acid* | | P | C_5_H_9_NO_4_ | | | 147.05321 | 0.83 | 0.55 | 1.97E-03 | | 2.13E-02 | | Amino acids, peptides, and analogues |
| 10 | N-Acetyllactosamine* | | P | C_14_H_25_NO_11_ | | | 383.14252 | 0.83 | 1.88 | 8.99E-06 | | 5.29E-04 | | Carbohydrates and carbohydrate conjugates |
| 11 | Acetylcarnitine* | | P | C_9_H_17_NO_4_ | | | 203.11592 | 0.86 | 0.61 | 3.11E-03 | | 3.04E-02 | | Fatty acid esters |
| 12 | 3,4-Dihydroxyhydrocinnamic acid* | | P | C_9_H_10_O_4_ | | | 164.04751 | 0.86 | 0.50 | 1.22E-03 | | 1.54E-02 | | Others |
| 13 | Aspartic acid* | | P | C_4_H_7_NO_4_ | | | 133.03755 | 0.86 | 0.45 | 7.95E-05 | | 2.05E-03 | | Amino acids, peptides, and analogues |
| 14 | Propionylcarnitine* | | P | C_10_H_19_NO_4_ | | | 217.13148 | 0.87 | 0.40 | 1.00E-05 | | 5.29E-04 | | Fatty acid esters |
| 15 | Proline* | | P | C_5_H_9_NO_2_ | | | 115.06336 | 0.92 | 0.18 | 3.44E-05 | | 1.19E-03 | | Amino acids, peptides, and analogues |
| 16 | N-Acetylneuraminic acid | | P | C_11_H_19_NO_9_ | | | 309.10566 | 1.05 | 0.45 | 4.58E-04 | | 7.82E-03 | | Carbohydrates and carbohydrate conjugates |
| 17 | Cytidine 5'-monophosphate* | | P | C_9_H_14_N_3_O_8_P | | | 323.05182 | 1.24 | 0.39 | 2.61E-06 | | 3.61E-04 | | Pyrimidine nucleotides |
| 18 | Glutathione* | | P | C_10_H_17_N_3_O_6_S | | | 307.08365 | 1.25 | 0.71 | 3.72E-03 | | 3.57E-02 | | Amino acids, peptides, and analogues |
| 19 | Tyrosine* | | P | C_9_H_11_NO_3_ | | | 181.07411 | 1.25 | 0.49 | 1.09E-03 | | 1.43E-02 | | Amino acids, peptides, and analogues |
| 20 | Adenosine 3'5'-cyclic monophosphate* | | P | C_10_H_12_N_5_O_6_P | | | 329.05292 | 1.36 | 2.23 | 9.94E-04 | | 1.33E-02 | | Purine nucleotides |
| 21 | Norleucine* | | P | C_6_H_13_NO_2_ | | | 131.09475 | 1.42 | 0.53 | 4.71E-03 | | 4.09E-02 | | Amino acids, peptides, and analogues |
| 22 | Phenylalanine* | | P | C_9_H_11_NO_2_ | | | 165.07916 | 2.14 | 0.55 | 1.39E-03 | | 1.71E-02 | | Amino acids, peptides, and analogues |
| 23 | Indoleacrylic acid* | | P | C_11_H_9_NO_2_ | | | 187.06348 | 4.21 | 0.54 | 9.05E-04 | | 1.25E-02 | | Others |
| 24 | Histidine* | | N | C_6_H_9_N_3_O_2_ | | | 155.06930 | 0.80 | 0.30 | 5.92E-04 | | 7.52E-03 | | Amino acids, peptides, and analogues |

RT, retention time; FDR, false discovery rate

Table S2 Differential metabolites identified in CCM metabolomic analysis

| No. | Metabolites | Ion mode | | | Formula | Molecular Weight | | RT (min) | Fold change | *P* value | FDR | Class |
| --- | --- | --- | --- | --- | --- | --- | --- | --- | --- | --- | --- | --- |
| 1 | Prolinamide | | P | C_5_H_10_N_2_O | | | 114.07940 | 0.72 | 1.21 | 4.00E-03 | 3.59E-02 | Amino acids, peptides, and analogues |
| 2 | Creatine* | | P | C_4_H_9_N_3_O_2_ | | | 131.06959 | 0.88 | 1.45 | 4.33E-03 | 3.79E-02 | Amino acids, peptides, and analogues |
| 3 | Glutamine* | | P | C_5_H_10_N_2_O_3_ | | | 146.06924 | 0.92 | 1.87 | 1.52E-08 | 1.17E-05 | Amino acids, peptides, and analogues |
| 4 | Pyridoxal | | P | C_8_H_9_NO_3_ | | | 167.05601 | 1.02 | 0.59 | 4.81E-03 | 4.04E-02 | Pyrimidine, Pyridines and derivatives |
| 5 | Niacinamide* | | P | C_6_H_6_N_2_O | | | 122.04800 | 1.26 | 9.24 | 5.42E-04 | 1.13E-02 | Pyrimidine, Pyridines and derivatives |
| 6 | Glutamic acid* | | P | C_5_H_9_NO_4_ | | | 147.05308 | 1.26 | 0.77 | 1.13E-03 | 1.65E-02 | Amino acids, peptides, and analogues |
| 7 | Valine | | P | C_5_H_11_NO_2_ | | | 117.07893 | 1.26 | 1.16 | 2.72E-03 | 2.85E-02 | Amino acids, peptides, and analogues |
| 8 | 4-Hydroxybenzaldehyde | | P | C_7_H_6_O_2_ | | | 122.03678 | 1.35 | 1.51 | 2.10E-04 | 5.51E-03 | Others |
| 9 | 3,4-Dihydroxyhydrocinnamic acid* | | P | C_9_H_10_O_4_ | | | 164.04743 | 1.35 | 1.55 | 2.09E-04 | 5.51E-03 | Others |
| 10 | 4-Acetamidobutanoic acid* | | P | C_6_H_11_NO_3_ | | | 145.07399 | 1.47 | 0.74 | 1.63E-03 | 2.12E-02 | Amino acids, peptides, andanalogues |
| 11 | Norleucine* | | P | C_6_H_13_NO_2_ | | | 131.09461 | 1.51 | 1.47 | 2.68E-03 | 2.83E-02 | Amino acids, peptides, andanalogues |
| 12 | 2'-O-Methylguanosine | | P | C_11_H_15_N_5_O_5_ | | | 297.10689 | 1.66 | 1.69 | 3.63E-04 | 8.17E-03 | Purine derivatives and purine nucleosides |
| 13 | Thymine | | P | C_5_H_6_N_2_O_2_ | | | 126.04294 | 2.01 | 4.02 | 2.85E-06 | 2.37E-04 | Pyrimidine, Pyridines and derivatives |
| 14 | Tryptophan* | | P | C_11_H_12_N_2_O_2_ | | | 204.08983 | 4.44 | 1.50 | 2.00E-03 | 2.35E-02 | Amino acids, peptides, and analogues |
| 15 | Phytosphingosine | | P | C_18_H_39_NO_3_ | | | 317.29297 | 7.93 | 0.17 | 1.17E-06 | 1.13E-04 | Others |
| 16 | Arginine* | | N | C_6_H_14_N_4_O_2_ | | | 174.11164 | 0.80 | 0.95 | 6.53E-03 | 3.61E-02 | Amino acids, peptides, and analogues |
| 17 | Galactose* | | N | C_6_H_12_O_6_ | | | 180.06356 | 0.91 | 3.31 | 2.31E-06 | 4.52E-04 | Carbohydrates and carbohydrate conjugates |
| 18 | 2-Furoic acid | | N | C_5_H_4_O_3_ | | | 112.01594 | 1.23 | 0.67 | 8.64E-04 | 8.35E-03 | Others |
| 19 | Oxoglutaric acid | | N | C_5_H_6_O_5_ | | | 146.02153 | 1.23 | 0.64 | 1.13E-03 | 9.80E-03 | Others |
| 20 | Xanthine* | | N | C_5_H_4_N_4_O_2_ | | | 152.03330 | 1.23 | 1.83 | 1.93E-05 | 1.21E-03 | Purine derivatives and purine nucleosides |
| 21 | 3-Hydroxymethylglutaric acid | | N | C_6_H_10_O_5_ | | | 162.05280 | 1.45 | 1.58 | 2.01E-03 | 1.43E-02 | Fatty acids and conjugates |
| 22 | Thymidine | | N | C_10_H_14_N_2_O_5_ | | | 242.09046 | 1.97 | 4.41 | 3.00E-06 | 4.52E-04 | Pyrimidine, Pyridines and derivatives |
| 23 | Docosahexaenoic acid | | N | C_22_H_32_O_2_ | | | 328.24046 | 14.99 | 2.08 | 2.50E-04 | 4.02E-03 | Fatty acids and conjugates |

CCM, cell culture medium; RT, retention time; FDR, false discovery rate

Table S3 Differential lipids identified in cell lipidomic analysis

| No. | Lipids | Formula | Fragments | RT (min) | m/z | Fold change | *P* value | FDR | Class |
| --- | --- | --- | --- | --- | --- | --- | --- | --- | --- |
| 1 | Cer (d17:1/24:1) | C_41_H_79_NO_3_ | [M+H] | 1.86 | 634.61327 | 0.34 | 4.05E-04 | 3.09E-02 | Cer |
| 2 | Cer (d18:0/16:0) | C_34_H_69_NO_3_ | [M+H] | 1.90 | 540.53502 | 0.56 | 4.74E-07 | 2.35E-04 | Cer |
| 3 | Cer (d18:1/16:0) | C_34_H_67_NO_3_ | [M+H] | 17.90 | 538.51937 | 0.41 | 2.71E-09 | 2.68E-06 | Cer |
| 4 | Cer (d18:1/18:0) | C_36_H_71_NO_3_ | [M+H] | 18.26 | 566.55067 | 0.32 | 8.41E-05 | 1.19E-02 | Cer |
| 5 | Cer (d18:1/20:0) | C_38_H_75_NO_3_ | [M+H] | 18.90 | 594.58197 | 0.26 | 1.64E-04 | 2.03E-02 | Cer |
| 6 | Cer (d18:1/24:1) | C_42_H_81_NO_3_ | [M+H] | 19.75 | 648.62892 | 0.36 | 5.90E-04 | 3.90E-02 | Cer |
| 7 | LPC (16:1) | C_24_H_48_NO_7_P | [M+H] | 20.06 | 494.32412 | 2.33 | 2.19E-04 | 2.04E-02 | LPC |
| 8 | LPC (18:4) | C_26_H_46_NO_7_P | [M+H] | 20.42 | 516.30847 | 2.59 | 2.56E-04 | 2.12E-02 | LPC |
| 9 | PC (20:4/13:1) | C_41_H_72_NO_8_P | [M+H] | 17.12 | 738.50683 | 1.38 | 2.79E-05 | 5.52E-03 | PC |
| 10 | PC (36:4) | C_44_H_80_NO_8_P | [M+H] | 18.40 | 782.56943 | 0.83 | 4.65E-04 | 3.29E-02 | PC |
| 11 | PS (18:0/20:4) | C_44_H_78_NO_10_P | [M+H] | 23.30 | 812.54361 | 0.57 | 4.62E-06 | 1.53E-03 | PS |
| 12 | TG (16:0/16:0/16:0) | C_51_H_98_O_6_ | [M+NH4] | 16.21 | 824.77017 | 0.57 | 2.18E-04 | 2.04E-02 | TG |
| 13 | TG (16:0/16:0/18:3) | C_53_H_96_O_6_ | [M+H] | 23.30 | 829.72797 | 0.72 | 6.91E-05 | 1.14E-02 | TG |
| 14 | TG (18:0/16:0/16:0) | C_53_H_102_O_6_ | [M+NH4] | 23.67 | 852.80147 | 0.58 | 2.72E-05 | 5.52E-03 | TG |
| 15 | TG (18:0/16:0/18:0) | C_55_H_106_O_6_ | [M+NH4] | 24.01 | 880.83277 | 0.60 | 2.26E-04 | 2.04E-02 | TG |
| 16 | CL (20:4/18:0/18:1/20:1) | C_85_H_154_O_17_P_2_ | [M-H] | 18.21 | 1509.06610 | 1.28 | 3.84E-04 | 4.68E-02 | CL |
| 17 | LdMePE (16:1) | C_23_H_46_NO_7_P | [M-H] | 1.83 | 479.30120 | 2.24 | 3.95E-04 | 4.68E-02 | LdMePE |

RT, retention time; Cer, ceramide; LPC, lysophosphatidylcholine; PC, phosphatidylcholine; PS, phosphatidylserine; TG, triglyceride; CL, cardiolipin; LdMePE, lysodimethylphosphatidylethanolamine
